# Psychological consequences of hospital isolation during the COVID-19 pandemic - research on the sample of polish firefighting academy students

**DOI:** 10.1007/s12144-021-01982-3

**Published:** 2021-06-28

**Authors:** Irena Walecka, Piotr Ciechanowicz, Klaudia Dopytalska, Agata Mikucka-Wituszyńska, Elżbieta Szymańska, Jacek Bogucki, Janusz Kock, Karolina Kułakowska, Wioletta Tuszyńska-Bogucka

**Affiliations:** 1 Dermatology Department, Centre of Postgraduate Medical Education / Central Clinical Hospital MSWiA, 137, Wołoska St., 02-507 Warsaw, Poland; 2 Department of Clinical Genetics, Medical University of Lublin, 1, Aleje Racławickie St., 20-059 Lublin, Poland; 3 Department of Anatomical, Histological, Forensic Medicine and Orthopedic Science, Sapienza University of Rome, 5, Piazzale Aldo Moro, 00-185 Rome, Italy; 4 Department of Human Sciences, University of Economics and Innovation in Lublin, 4, Projektowa St., 20-209 Lublin, Poland

**Keywords:** Post-traumatic stress disorder, Post Covid-19 traumatic stress syndrome, Coronavirus, SARS-CoV-2, Depression

## Abstract

Currently, a very important thread of research on COVID-19 is to determine the dimension of the psychopathological emotional reactions induced by the COVID-19 pandemic. A non-experimental online research project was designed to determine the predictors of the severity of psychopathological symptoms, such as depression and PTSD symptoms, and the nature of the feedback mechanism between them in groups of men, remaining in hospital isolation due to infection and at-home isolation during the COVID-19 epidemic. The presence of symptoms of depression, post-traumatic stress disorder (PTSD) and a sense of threat due to the pandemic were assessed using the following screening tests: IES-R by Weiss and Marmar, PHQ-9 by Spitzer et al., and a self-constructed sliding scale for assessing COVID-19 anxiety. The study was carried out on a group of 57 firefighting cadets, hospitalized in a COVID-19 isolation room (M_age_ = 23.01), staying in isolation due to SARS-CoV-2 virus infection and a control group of 57 healthy men (M_age_ = 41.38) staying at home during quarantine and national lockdown. COVID-19 pandemic causes many psychopathological reactions. The predictive models revealed that the predictors of symptoms of PTSD in isolated patients included depression and the experienced sense of COVID-19 threat resulting from the disease, while in the control group the symptoms of depression were the only predictor of PTSD. PTSD experiences are usually associated with depression. It may also be a form of the re-experiencing process or the effect of high affectivity, indirectly confirmed by the participation of hyperarousal in the feedback loop. Our findings highlight the importance of mental health aspects in patients treated during the COVID-19 pandemic. The COVID-19 pandemic requires social distancing, quarantine and isolation, which may cause psychopathological symptoms not only in affected people, but also in the general population. Moreover, the need for greater psychological support can be emphasized for both: the sick and the general population.

## Introduction

*“The world has changed dramatically over the last eight months”*

*(*Asmundson & Taylor, 2020a, 2020b*).*

In December 2019, several cases of pneumonia of unknown etiology were diagnosed in China. In January 2020 it was announced that the symptoms were caused by a new coronavirus - *Severe Acute Respiratory Syndrome Coronavirus 2* (SARS-CoV-2), causing the symptoms of the *Coronavirus Disease 2019* (COVID-19) (Giwa et al., 2020; Mohammadi et al., 2020). Due to the very rapid spread of the virus around the world, the World Health Organization (WHO) declared a global pandemic. To date, 96,267,473 have been infected, and 2,082,745 have died in the course of the disease (as of January 22, 2021) (World Health Organization, 2021). SARS-CoV-2 infection is more commonly diagnosed in men and mainly affects people over 40 (Guan et al., 2020; Huang et al., 2020). The virus affects the respiratory tract, and the nostrils are the gateway through which the infection may spread. After an incubation period of 2–14 days, the patient manifests the first symptoms of the disease (Li et al., 2020). The most common ones being fever, malaise/fatigue, cough, tightness in the chest and shortness of breath. Cough is usually non-productive. Occasionally, the symptoms include smell and/or taste disorders, gastrointestinal symptoms (abdominal pain, nausea, vomiting, diarrhea) or skin lesions (Chen et al., 2020; Ciechanowicz et al., 2020; Jiang et al., 2020; Piva et al., 2020). In summary, it seems that describing the statistics of the disease, that vary on a daily basis, is not as important as the fact that the number of SARS-CoV-2 infections continues to increase, as does the number of deaths caused by it (Asmundson & Taylor, 2020c), which is a very serious research problem for both medical sciences and psychology.

According to WHO data (World Health Organization, 2020) approximately 80% of patients with a confirmed COVID-19 infection do not require inpatient care due to mild or asymptomatic course of the disease. Moreover, such a course of infection most often occurs in children and young adults who need to be isolated from the rest of the society (Brooks et al., 2020; Garg et al., 2020).

The rapid spread of SARS-CoV-2 infection and the lack of the preparation of healthcare systems in various countries led to a global panic among governments, institutions and ordinary citizens (Rahman et al., 2020). Currently, a very important thread of research on COVID-19 is to determine the dimension of the psychopathological emotional reactions induced by the COVID-19 pandemic, especially in the face of the announcements of further waves of the disease.

The research team of Asmundson and Taylor, claimed that the coronavirus pandemic would inflict profound psychological effects on many individuals, and the resulting disorders would have the nature of the post-traumatic stress disorder (PTSD) (Asmundson & Taylor, 2020a, 2020b; Taylor et al. 2020). Eventually, their concerns were confirmed by research. In the general population, PTSD-type psychopathological reactions affect up to 10% of the population (Giourou et al., 2018) and are mainly associated with experiencing events such as catastrophes or sudden threats. The COVID-19 pandemic is a unique phenomenon, unprecedented thus far (Curkovic et al., 2020) It seems to be a traumatic experience for many groups (Sood, 2020; Torales et al., 2020; Yuan et al., 2021).

The results of previous scientific studies (Horn & Feder, 2018; Ibbotson, 1999) suggested that previous stressful experiences, isolation from people who are close and improper resilience may increase the risk of depressive episodes and PTSD reactions in a subject. In many of the studies during the first wave, which empirically determined the severely stressful nature of the SARS-Cov-2 virus infection associated with numerous neuropsychiatric disorders, it has been postulated to undertake research both in groups of ill subjects, essential service workers, and in the healthy population, also heavily burdened with pandemic stress (Troyer et al., 2020), which is what our work has been assessing.

### Aims and Hypotheses

The aim of our study was to determine the increase in the risk of depressive reactions and/or PTSD in the group of the firefighting cadets in isolation due to SARS-CoV-2 infection compared to a corresponding group of healthy people.

Based on the findings of Ibbotson (Ibbotson, 1999), we expected that the measurements would reflect the elevated levels of depression and PTSD responses in hospitalized firefighting cadets due to their possible exposure to previous stressful events, experience of current health hazards, and the setting of hospital isolation. High levels of depression are associated with anxiety disorders, such as PTSD, so they may also be expected to show the signs of despondency and achieve higher scores on the depression test. The objective of the intergroup comparison was to test the hypothesis that firefighters infected with SARS-CoV-2 virus and in hospital isolation were more susceptible to acute post-traumatic stress and depression in comparison with healthy people.

In our research we attempted to determine whether:

1. the disease and remaining in isolation constitute traumatic stressors, and, if so, which elements dominate in patients’ experiences;
2. the occupation of an essential service worker (firefighter) is a protective factor in the intensification of the psychopathological PTSD-type reactions, depression and anxiety;
3. the epidemiologic situation is a burden for healthy people who have not experienced a situation of an immediate threat, such as the infection with the virus?

The assumed method allowed us to meet the demand for mental health examinations in the most important groups, which include: (1) Primary Group – General Population, (2) Essential Service Workers, (3) Students and Teachers, and (4) Confirmed COVID-19 Victims (Giourou et al., 2018). Our work presents a study of a special group of COVID-19 patients, i.e. young firefighters (cadets) hospitalized in an isolation room after being infected with the SARS-CoV-2 virus. This specific group has been selected for the study due to the reported particular exposure to occupational stress (Ibbotson, 1999), which may increase the susceptibility to stress related to the illness and the stay in an isolation room. The indicators of psychopathological reactions which they showed during the 14-day isolation period were compared to the psychopathological reactions of healthy people remaining not in quarantine, but in the conditions of home isolation.

## Methods

### Participants and Procedure

The group of patients in isolation included the students (cadets) of a firefighting academy. In the initial period of the pandemic, they spent time in barracks, allowing for the disease to be contracted collectively. Later, they were placed in isolation either in solitary or double rooms. The tests were performed during the second week of isolation. The study included a group of 57 young men (M_age_ = 23.01), the cadets of the firefighting academy, without any comorbidities, staying in isolation due to SARS-CoV-2 virus infection. The majority of patients did not present with any symptoms of COVID-19, while 18 (31.57%) men had a mild course of the disease. The most common symptoms were olfactory disorders (*n* = 11–19.29%), taste disorders (*n* = 8–14.03%) and non-specific chest pain (n = 8–14.03%).

The control group consisted of 57 male volunteers (M_age_ = 41.38), who remained under quarantine conditions at home, during a general lockdown in Poland. The participation in the research did not involve any financial compensation. The remaining socio-demographic characteristics are presented in Table 1.

**Table 1:** Socio-demographic characteristics of the groups included in the study

Variable	Group				p	Eta Squared/V Cramer
	patients		control			
**Age** (M ± SD)	23.01 ± 3.87		41.38 ± 12.82		<0.0001	.545
**Place of residence**	N	%	N	%	<0.0001	.500
Countryside	28	49.12	8	14.03		
Country town (up to 25 thousand inhabitants)	10	17.54	4	7.01		
Small town (25–50 thousand inhabitants)	5	8.77	4	7.01		
Mid-sized town (50–300 thousand inhabitants)	4	7.01	5	8.77		
City (above 300 thousand inhabitants)	10	17.54	36	63.15		
**Educational level**					<0.0001	.740
Secondary and achieving higher education	49	85.96	11	19.29		
Higher education and currently studying	7	12.28	5	8.77		
Primary education	–	–	1	1.75		
Higher education	1	1.75	30	52.63		
Secondary education	–	–	10	17.54		
**Marital status**					<0.0001	.633
Single	55	96.49	21	36.84		
Married	2	3.50	30	52.63		
Divorced	–	–	5	8.77		
Separated	–	–	1	1.75		
**Material status**					0.372	.193
Very good	3	30	7	12.28		
Good	29	50.88	33	57.89		
Average	21	36.84	14	24.56		
Low	3	5.26	3	5.26		
Very low	1	1.75	0	–		

The studied group is relatively small, but it was a unique and comprehensive group (cadets of one school, who were all infected in the first days of the pandemic and national quarantine, and have been hospitalized together), therefore it was decided to retain its original character. It also meets the basic criteria of a group whose results can be statistically analyzed (significance of differences analysis and regression analysis).

### Measures

***IES-R*** of Weiss and Marmar in the Polish adaptation of Ogińska-Bulik and Juczyński (2009) is used to measure *PTSD* symptoms. It consists of 22 statements describing the symptoms of stress experienced in the last 7 days in relation to the experienced traumatic event. Ratings are made on a 5-point Likert scale (0–4). It is used to determine the current, subjective feeling of discomfort related to the specific event that has occurred. It addresses the three aspects of PTSD: 1) *Intrusion,* expressing recurring images, dreams, thoughts or perceptual experiences related to the trauma; 2) *Hyperarousal*, characterized by increased vigilance, impatience, difficulty focusing, and 3) *Avoidance*, manifested by efforts to get rid of thoughts, emotions or conversations related to the trauma. The reliability of the scale was assessed by estimating its internal consistency and absolute stability. The internal consistency, assessed on the basis of Cronbach’s alpha-factor, is 0.92 for the entire scale, with the individual values for *Intrusion, Hyperarousal and Avoidance* being 0.89, 0.85 and 0.78, respectively. Most of the statements correlate above 0.60 with the overall score of the scale (Juczyński & Ogińska-Bulik, 2009).

The **PHQ-9** by Spitzer et al. (Spitzer et al., 1999), in the Polish adaptation of Kokoszka et al. (Kokoszka et al., 2016) consisting of 9 core questions and one supplementary question, was the tool of choice for the measurement of *depression*. The main questions concern the symptoms of depression included in the DSM-IV diagnostic criteria. The respondent marks the answers on a scale from 0 to 3, depending on the frequency of occurrence of a given symptom in the last two weeks. The PHQ-9 is characterized by good internal consistency, since Cronbach’s alpha-factor is equal to 0.70. In the Polish validation studies of the questionnaire, a value from 6 to 12 was adopted as the optimal cut-off point for the diagnosis of depression upon screening (Kokoszka et al., 2016; Tomaszewski et al., 2011). In our research, however, we adopted a universal cut-off point of 10, as proposed by Manea et al. (Manea et al., 2012), due to the best psychometric values ​​of the scale (sensitivity – 80%, specificity – 92% in detecting a depressive episode) as well as the age profile of the studied group. The Polish version of the PHQ-9 has adequate psychometric properties and is an effective screening tool for depression in people aged 18–60.

Furthermore, the measurement of the sense of threat in the setting of the COVID-19 pandemic was done using a self-designed tool: a numerical sliding scale. The simple pictorial method was modelled basing on popular pain assessment scales. A scale from 0 to 10 was used by the respondents to assess the severity of the sense of threat experienced in connection with their situation (in the patient group it was related to the presence of the disease, in the group of healthy people – the possibility of acquiring the disease). This short and simple tool was modeled on the recognized method of measuring the intensity of pain, and the decision to use it was dictated by the need of the moment (no tool for measuring pandemic anxiety has been developed so far). It seems that the pandemic fear is a completely new construct (Arora et al., 2020), which justifies attempts to use new methods of its measurement, as the old ones do not apply in this particular situation. During the first wave of the pandemic, in the spring of 2020, there was no specific tool adapted for measuring the fear of infection with the coronavirus type FCV-19S (Ahorsu et al., 2020), as it was only developed at the end of 2020. In addition, the condition of the isolated patients and their burden of undergoing a high number of various types of tests, justifies the use of such brief tools, if only possible.

All of the methods had been developed in the form of an online set and made available to the participants of the study. The duration of the study was not limited. All tools were dedicated (instruction manual) to the COVID-19 pandemic situation.

### Statistical Analysis

Descriptive analyses and clinical classification were performed. To examine associations between the psychopathological emotional reactions induced by the COVID-19 pandemic, multiple regressions were performed in IBM Statistics SPSS 26.0. Three regression analyzes were performed: prediction of PTSD symptoms (model 1), depression (model 2) and fear of COVID-19, taking into account the interrelationships between the studied variables.

## Results

Firstly, the authors assessed the obtained results according to the criteria of clinical diagnosis (Table 2). Regarding the IES-R result (PTSD severity), it is advised to adopt a borderline value, from which the results of the examined person may be considered as indicative of the presence of stress after experiencing trauma. Taking the mean score of 1.5 of the overall scale as the cut-off value, a score above this point may be treated as a PTSD index. With a more restrictive approach, justified by the current criteria for diagnosing PTSD, a diagnosis of PTSD could only be suspected in those individuals who score above the intercept (> 1.5) in each of the three aspects (intrusion, hyperarousal, avoidance). The analysis showed that 22.81% of the patients in isolation confirmed the experience of trauma, revealing a high level of psychological discomfort related to the COVID-19 pandemic situation (when accepting the average score of 1.5 of the general scale index as the borderline value). PTSD symptoms were shown by 26.31% of healthy people with a more restrictive approach undertaken (when the result of each subscale exceeded the value of 1.5). Out of 57 patients, 7.01% achieved the criteria for the clinical diagnosis of PTSD (compared to 17.54% in the group of healthy subjects). This criterion, i.e. a score above 1.5 points in each of the 3 aspects, seems to reflect the scale of the phenomenon more accurately (Juczyński & Ogińska-Bulik, 2009).

**Table 2:** The occurrence of psychopathological reactions in the studied groups according to each clinical criterion

	Total score of PTSD symptoms > 1.5				Symptoms of Intrusion, Hyperarousal, Avoidance > 1.5				Symptoms of Depression > 10			
	patients		control		patients		control		patients		control	
	N	%	N	%	N	%	N	%	N	%	N	
Failure to meet the scoring criterion	44	77.19	42	73.69	53	92.99	47	82.46	45	78.93	42	73.66
Scoring criterion met	13	22.81	15	26.31	4	7.01	10	17.54	12	21.04	15	26.31

A similar clinical diagnosis was made when analyzing the severity of depression symptoms. The authors of the Polish version of the tool searched for optimal cut-off points, thus increasing the psychometric properties in relation to the situation, where the recommended values ​​were adopted in advance. With the adoption of the cut-off point at 10, the data indicated that the percentage of people showing the symptoms of depression was 21.03% in the patient group and 26.31% among the healthy subjects.

The next step involved assessing whether the studied groups differed statistically in the intensity of the psychopathological symptoms studied (Table 3). Due to the distribution of the studied variables, the Mann-Whitney U test was used in order to assess the significance of differences.

**Table 3:** The significance of differences in the intensity of PTSD and depression symptoms in the studied groups

	M		SD		p	Eta-squared
	patients	control	patients	control		
Symptoms of Intrusion	5.15	8.80	5.12	7.54	**0.006**	**.006**
Symptoms of Hyperarousal	6.50	7.71	5.56	6.73	0.491	–
Symptoms of Avoidance	6.91	9.10	5.10	6.07	0.092	–
Total score of PTSD symptoms	18.57	25.63	14.14	18.81	0.076	–
Symptoms of Depression	5.35	5.36	5.35	5.84	0.918	–
Sense of COVID threat symptoms	3.43	2.54	2.19	1.18	0.087	–

The comparison of the studied groups did not reveal statistically significant differences in the intensity of psychopathological reactions: hyperarousal, avoidance, depression and anxiety. The only significant difference concerned the intensity of intrusion (recurring images, dreams, thoughts or perceptual impressions related to trauma), which was significantly higher (Z = −2.69758; *p* = 0.006) in healthy people staying in home quarantine.

Three multiple regression analyses introducing information on the severity of PTSD (model 1), depression (model 2) and the sense of threat (model 3) were used to assess their predictive role in the severity of other psychopathological reactions in the study groups. The results are presented in Table 4 (the table contains only statistically significant results.^1^ The predictor for PTSD among hospitalized COVID-19 patients in isolation were both: depression and the experienced sense of COVID-19 threat resulting from the disease. As regards healthy subjects, PTSD was induced only by depression. However, these results concern only one element of PTSD, i.e. hyperarousal (increased alertness, impatience, difficulty in concentrating). In turn, only hyperarousal was the predictor of depression, both in the group of isolated patients and in healthy people (Table 5).

**Table 4:** Regression analysis – statistically significant results

SYMPTOMS OF PTSD (model 1)						
patients						
	b*	SE of b*	b	SE of b	t(54)	p
constant term			5.933	3.174	1.868	0.067
Symptoms of Depression	0.525	0.109	1.389	0.290	4.784	<**0.001**
Symptoms of Sense of COVID-threat	0.235	0.109	1.515	0.708	2.140	**0.036**
control						
constant term			15.501	4.914	3.154	**0.002**
Symptoms of Depression	0.637	0.105	2.052	0.338	6.059	<**0.001**
SYMPTOMS OF DEPRESSION (model 2)						
patients						
constant term			2.229	1.238	1.800	0.077
Symptoms of Hyperarousal	0.587	0.223	0.565	0.214	2.630	**0.011**
control						
constant term			1.179	1.506	0.782	0.437
Symptoms of Hyperarousal	0.835	0.233	0.725	0.202	3.579	<**0.001**

**Table 5:** Regression analysis – comprehensive table

	patients						control					
SYMPTOMS OF PTSD (model 1)												
	b*	SE of b*	b	SE of b	t(54)	p	b*	SE of b*	b	SE of b	t(54)	p
constant term			5.933	3.174	1.868	0.067			15.501	4.914	3.154	**0.002**
Symptoms of Depression	0.525	0.109	1.389	0.290	4.784	<**0.001**	0.637	0.105	2.052	0.338	6.059	<**0.001**
Symptoms of Sense of COVID-threat	0.235	0.109	1.515	0.708	2.140	**0.036**	−0.021	0.105	−0.349	1.677	−0.208	0.835
SYMPTOMS OF DEPRESSION (model 2)												
constant term			2.229	1.238	1.800	0.077			1.179	1.506	0.782	0.437
Symptoms of Intrusion	0.087	0.219	0.091	0.229	0.397	0.692	0.077	0.243	0.059	0.188	0.317	0.752
Symptoms of Hyperarousal	0.587	0.223	0.565	0.214	2.630	**0.011**	0.835	0.233	0.725	0.202	3.579	<**0.001**
Symptoms of Avoidance	−0.081	0.147	−0.085	0.155	−0.550	0.584	−0.267	0.133	−0.257	0.128	−2.004	0.050
Symptoms of Sense of threat	−0.052	0.116	−0.127	0.283	−0.448	0.655	0.032	0.094	0.159	0.467	0.342	0.733
SYMPTOMS OF SENSE OF COVID THREAT (model 3)												
constant term			2.510	0.516	4.859	<0.001			2.444	0.295	8.281	<0.001
Symptoms of Intrusion	−0.093	0.261	−0.040	0.111	−0.359	0.720	−0.550	0.350	−0.086	0.054	−1.572	0.121
Symptoms of Hyperarousal	0.202	0.281	0.079	0.111	0.719	0.474	0.454	0.377	0.079	0.066	1.205	0.233
Symptoms of Avoidance	0.261	0.172	0.112	0.074	1.512	0.136	0.094	0.202	0.018	0.039	0.465	0.643
Symptoms of Depression	−0.073	0.164	−0.030	0.067	−0.448	0.655	0.069	0.203	0.014	0.041	0.342	0.733

**Fig. 1:**
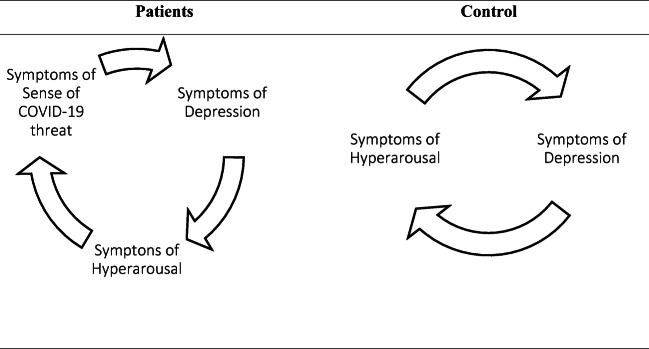
Possible feedback loops of psychopathological reactions in the studied groups

The analysis showed that the sense of threat in the group of patients might play a role of a trigger of subsequent psychopathological reactions, such as depression and hyperarousal. The factor did not play such a crucial role in the group of healthy people (Fig. 1).

## Discussion

The results show that the disease and the stay in the isolation room did not trigger a significantly greater intensity of psychopathological reactions (PTSD, depression and a sense of threat) in the affected subjects when compared to healthy subjects remaining in home quarantine. The patients in the isolation rooms also showed a significantly lower intensity of intrusion compared to the healthy subjects remaining in home isolation. It means that the general population seems to be more vulnerable to intrusive experiences, such as intrusive memories of events, repeated disturbing dreams, recreating or feeling a worst-case scenario of specific events, anxiety caused by internal or external triggers of trauma, or a physiological response to internal/external trauma triggers (Giourou et al., 2018).

### Occupational Resilience?

It seems that the hospitalized firefighters showed a relatively high resistance in the conditions of the presence of the disease and isolation. They did not differ from the healthy sample with regard to the intensity of psychopathological reactions, such as depression and anxiety, as well as the elements of PTSD, avoidance and hyperarousal. They also showed a significantly lower intensity of intrusion. It proves their good personal adaptation skills and, moreover, the suitability of the selection criteria applied by the academy while recruiting candidates. Conversely, it may be the effect of a well-functioning isolation room, which resulted in minimizing the negative emotional reactions of patients. However, taking into account that the literature reports the occurrence of serious neuropsychiatric effects due to the SARS-CoV-2 virus infection, not only the direct and indirect neuropsychiatric effects of viral infection on the mental health status of the patients should be taken into account (Asmundson & Taylor, 2020a, b, c), but also the effects of prolonged isolation on the well-being of healthy people.

### Feedback Loops

The results support the conclusion that PTSD experiences are usually associated with depression. It may also be a form of the re-experiencing process or the effect of high affectivity (Flory & Yehuda, 2015), indirectly confirmed by the participation of hyperarousal in the feedback loop. In isolated patients, the triggering mechanism of psychopathological reactions in the form of hyperarousal (a PTSD element) seems to be the threat of the SARS-CoV-2 infection. The loop has a different form in healthy people: the feeling of being in danger is not a predictor of PTSD or depression.

It is worth emphasizing that the presence of hyperarousal is the only element of PTSD activated in the situation of reacting to the disease and isolation. Therefore, it seems that in PCTSS one should mainly consider symptoms such as difficulty falling asleep or maintaining sleep, irritability or outbursts of anger, difficulty concentrating, excessive vigilance or exaggerated responses to stimuli as a result of the pandemic, not only in patients, but also in the general population (Giourou et al., 2018; Steardo Jr. et al., 2020).

### The Prevalence of Psychopathological Reactions

Our results indicate that the COVID-19 pandemic is a factor causing multiple psychopathological reactions (21.04% in the group of patients and 26.31% in the group of healthy people showed the clinical symptoms of depression, about 7% of patients had the symptoms of PTSD, compared to 17.5% of healthy people). The results of the clinical analysis correspond with the results obtained in other countries, demonstrating high rates of psychological problems, such as acute post-traumatic stress or depression, among people exposed to trauma resulting from the COVID-19 epidemic (Cenat et al., 2020; Choi et al., 2020; Consolo et al., 2020; Fekih-Romdhane et al., 2020; Gualano et al., 2020; Luo et al., 2020; Salari et al., 2020; Steardo Jr. et al., 2020; Xiao et al., 2020). Thus, depression is a more common problem in isolated patients, and they are less affected by PTSD. Importantly, stress affects very healthy people, which confirms the importance of psychoeducation and psychosocial support in preventing and combating “epidemic anxiety” (the fear of a pandemic) not only among sick people but also in the general non-affected population (Vostanis & Bell, 2020). It may also be concluded that the psychological effects of the COVID-19 pandemic in the studied group are dimensionally comparable to the effects of traumatic events such as catastrophes, critical incidents or situations of serious health or life threat, although they are of a slightly different nature. Therefore, the new term Post Covid-19 Traumatic Stress Syndrome – PCTSS (Giourou et al., 2018) is justified in characterizing emotional states during the COVID-19 pandemic.

### Limitations and Future Directions

Our study is not free from disadvantages, mostly resulting from the *nature of the group and the measurement methods*. Cases and controls were not matched for age, which is an important confounding factor in determining mental health outcomes. The group of surveyed firefighters is a group of young people, who usually knew each other from their studies, some of the subjects had higher education, which may contribute to specific protective factors. Moreover, the mechanisms of immunity could be strengthened by experiencing the disease as a group. The fact that the subjects had constant contact with each other (via internet) might also lead to a social immunity resource. In future research it is also worth considering the role of other SES variables (profession, marital status, education level, living environment) that distinguish the studied groups in moderating the pandemic stress effect. Interestingly enough, the studied groups did not differ in their material status, suggesting a different role of this resource in protection against the pandemic stress. The tests should additionally be performed in groups of women. The measurement methods, mainly consisting of short, screening tools, may have also been a limitation. However, mainly due to the fact that the group of patients in the isolation room underwent various types of tests, it was deemed a necessity. Despite the indicated limitations, the results seem to carry an applicable value in the form of the explanation of the specifics of the psychopathological feedback loop mechanism and indications for assistance in such situations. It is also worth noting that the group of patients in the isolation room undergoing the study had a unique character, perhaps allowing for the creation of a specific community even in hospital conditions, and also a distinct career profile. The results indicate some resilience resources in a pandemic setting (Vagni et al., 2020), especially in the case of PTSD symptoms, compared to the control group consisting of various different people who voluntarily decided to participate in the study. Thus, the interpretative perspective was expanded due to the dissimilarity of the studied groups.

As regards future research projects, it seems necessary to monitor patients in terms of the persistence of the identified symptoms and their further coping with the experienced trauma. It is also worth including the diagnosis of dissociative symptoms, which constitute an additional criterion in the acute stress syndrome. Psychoeducation is the most important strategy of coping with fears and a sense of threat: providing patients with adequate information about the cause, mechanism of anxiety, as well as the possibility to join the treatment process, and, thus, causing an increase in its effectiveness. In many cases, cognitive-behavioral therapy is also recommended, which, apart from teaching correct behaviors, focuses on correcting inappropriate attitudes and beliefs as well as maladaptive ways of thinking about the threat of the disease and the related situation (Brooks et al., 2020; Xiang et al., 2020). Perhaps, in a setting of isolation rooms, launching an online help group should be considered as a method of additional support in dealing with the trauma due to the burden of the disease and the isolation conditions. New treatment strategies are also needed to address the unique psychological and biological aspects of accompanying comorbidities. It seems that the direction of further research should focus on determining the severity of other neuropsychiatric symptoms, described in literature as the pandemic effect (Kang et al., 2020; Troyer et al., 2020).

## Conclusions

1. The results suggest that the hospitalized group consisting of essential service workers showed high resistance to the burden of the disease and the resulting hospital isolation. The patients in isolation rooms did not show a significantly higher intensity of symptoms of PTSD, depression nor the sense of threat associated with COVID-19 infection, compared to the healthy subjects. It may prove both a high level of resistance to difficult situations presented by the hospitalized firefighters, as well as proper functioning and conditions in the isolation room.
2. The analysis showed a different configuration of the predictors of psychopathological reactions in the studied groups, where the sense of threat played a different role. As regards this symptom, psychological work seems to be an important aspect of psychological assistance when dealing with the situation of illness and remaining in an isolation room.
3. The COVID-19 pandemic triggered a significant number of psychopathological reactions in the study groups, mainly depressive and, to a lesser extent, PTSD. The study also shows that it is not necessary to be ill in order to experience a psychopathological reaction, since healthy people also showed a severe aggravation of such reactions, sometimes even more marked than isolated patients (PTSD – intrusions). They also more commonly experienced the clinical symptoms of PTSD. It suggests the necessity to diagnose the depth of the disorders and to use additional methods of treating possible accompanying mental disorders, both in the group of patients and in the control sample.

## Data Availability

All data generated or analyzed during this study are included in this published article [and its supplementary information files].

## Footnotes

1 For the complete regression analysis results, see the supplementary material.
